# Episodes of care in a primary care walk-in clinic at a refugee camp in Germany – a retrospective data analysis

**DOI:** 10.1186/s12875-020-01253-3

**Published:** 2020-09-21

**Authors:** Jan Hendrik Oltrogge, Ingmar Schäfer, Dana Schlichting, Martin Jahnke, Anja Rakebrandt, Susanne Pruskil, Hans-Otto Wagner, Dagmar Lühmann, Martin Scherer

**Affiliations:** 1 Department of General Practice and Primary Care, University Medical Center Hamburg-Eppendorf (UKE), Martinistraße 52, 20246 Hamburg, Germany; 2 Local Health Authority – Altona, Bahrenfelder Straße 254-260, 22765 Hamburg, Germany

**Keywords:** Asylum seeker, Refugee, Public health, Primary healthcare centers, Health systems, Care planning

## Abstract

**Background:**

From 2015 to 2016 Germany faced an influx of 1.16 million asylum seekers. In the state of Hamburg Primary Care walk-in clinics (PCWC) were commissioned at refugee camps because the high number of residents (57,000 individuals) could not be provided with access to regular healthcare services. Our study aims were (1) to describe the utilization of a PCWC by camp residents, (2) to compare episodes of continuous care with shorter care episodes and (3) to analyse which diagnoses predict episodes of continuous care in this setting.

**Methods:**

A retrospective longitudinal observational study was conducted by reviewing all anonymized electronic medical records of a PCWC that operated from 4th November 2015 to 22nd July 2016 at a refugee camp in Hamburg. Episodes of care (EOC) were extracted based on the international classification of primary care-2nd edition (ICPC-2). Outcome parameters were episode duration, principal diagnoses, and medical procedures.

**Results:**

We analysed 5547 consultations of 1467 patients and extracted 4006 EOC. Mean patient age was 22.7 ± 14.8 years, 37.3% were female. Most common diagnoses were infections (44.7%), non-communicable diseases (22.2%), non-definitive diagnoses describing symptoms (22.0%), and injuries (5.7%). Most patients (52.4%) had only single encounters, whereas 19.8% had at least one EOC with a duration of ≥ 28 days (defined as continuous care). Several procedures were more prevalent in EOC with continuous care: Blood tests (5.2 times higher), administrative procedures (4.3), imaging (3.1) and referrals to secondary care providers (3.0). Twenty prevalent ICPC-2-diagnosis groups were associated with continuous care. The strongest associations were endocrine/metabolic system and nutritional disorders (hazard ratio 5.538, *p* < 0.001), dermatitis/atopic eczema (4.279, p < 0.001) and psychological disorders (4.056, p < 0.001).

**Conclusion:**

A wide spectrum of acute and chronic health conditions could be treated at a GP-led PCWC with few referrals or use of medical resources. But we also observed episodes of continuous care with more use of medical resources and referrals. Therefore, we conclude that principles of primary care like continuity of care, coordination of care and management of symptomatic complaints could complement future healthcare concepts for refugee camps.

## Background

In 2015 and 2016, the European Union (EU) faced a significant increase in the number of asylum seekers and Germany was the primary destination for asylum seekers in the EU [1, 2]. Due to a housing shortage in the federal state of Hamburg, the local government approved the temporary conversion of non-residential facilities into refugee camps, so-called First Reception Centres (FRC) where up to 1600 refugees were accommodated at a single location [3]. This overwhelming high number led to extended processing times of asylum applications. Many FRC residents could not be provided timely with a National health insurance registration, which is needed for the utilization of health care services in Germany. To ensure medical care for FRC residents, the local health authorities of Hamburg commissioned a primary care walk-in clinic (PCWC) in one of the biggest FRC in Hamburg. The GPs at the PCWC were provided with basic medical equipment and online access to formal interpreters. But the question remained to what extend such a low-threshold primary care service with the ability to provide continuous primary care would be utilized by the residents and whether their healthcare needs could be adequately addressed. Various reports suggested that the healthcare needs of asylum seekers differ considerably from the general population [4]. The prevalence of mental health disorders is considered to be higher [5]. In Germany the utilization of secondary care services, hospitalization rates and healthcare expenditures regarding asylum seekers were reported to be above-average [6]. These observations led to claims for a more integrated and more individualized health care provision for refugees in Europe [7]. However, we noted that most studies that investigated healthcare in refugee camps had a cross-sectional approach and evaluated the prevalence of specific health conditions (e.g., psychiatric conditions or communicable diseases) or focused on selected populations such as unaccompanied minors [8–14]. We concluded that more knowledge is needed on the provision of continuous primary care for asylum seekers since continuous care is considered an important contributor to quality-of-care and patient satisfaction [15, 16] .

The aims of the study were (1) to describe the utilization of the PCWC as indicated by treatment rates, disease spectrum and the duration of care episodes; (2) to compare episodes of continuous care with shorter care episodes regarding initiated medical procedures and (3) to analyse which diagnoses predict the need for continuous care in a population of refugee camp residents.

## Methods

The study took place at a PCWC that was localized at one of the biggest FRCs in the city of Hamburg. The clinic was operated for 9 months by GPs from the Department of General Practice and Primary Care of the University Medical Center Hamburg-Eppendorf (UKE) during the peak influx of refugees to Germany. In a comprehensive approach, all electronic medical records (EMR) of the PCWC were coded with the *international classification of primary care - second edition* (ICPC-2) to compose episodes of care (EOC) for further analysis.

### Setting

One of the biggest FRC for refugee seekers in the city of Hamburg was a converted former hardware store with a housing capacity for up to 1600 residents (“*Zentrale Erstaufnahme – Am Rugenbarg”; FRC-Rugenbarg*). It was managed by the German Red Cross District Association. A PCWC was established in a partnership between the local health authorities of Hamburg and the Department of General Practice and Primary Care of the UKE. From the 4th of November 2015 until the decommissioning of the *FRC-Rugenbarg* on 22nd of July 2016, the PCWC operated from Monday to Friday for 8 h a day. The GPs were provided with an office and a treatment room equipped with a live video translation service in a reconstructed freight container [17]. Patients could be examined and treated in terms of basic general medical treatment, wound treatment and mild injuries. GPs did not have to initiate preventative measures since all asylum seekers underwent a medical baseline examination which included basic immunization protocols as well as screening for tuberculosis and other infectious diseases of public health concern. However, GPs took care of the completion of immunizations in their consultations. When there was a suspicion of an infectious disease GPs could consult the department of infectiology of the University Medical Center. Common medications could be handed over out of a medicine cabinet on-site. Additionally, GPs could issue prescriptions for less frequent medications that were accepted by local pharmacies without any additional costs for the patients. When imaging and laboratory testing were deemed to be medically necessary, patients were sent to the primary care outpatient clinic at the University Medical Center. All requested investigations that complied with the catalogue of the German statutory health insurance could be performed without any additional costs. In order to refer a patient to a specialist or to hospital care, GPs issued a letter of referral and a standard form that equated to an assumption of costs for secondary care providers. First-aid was provided at any time by the Red Cross District Association. In case of a suspected life-threatening emergency, the regular emergency medical services of the city of Hamburg were notified.

### Data source

Electronic medical records (EMR) at *FRC-Rugenbarg* were generated on a laptop with a secure internet connection and stored in a cloud-based EMR-database (‘BASIS4Refugees’; Medisoft, Hamburg). Therefore, no hand-written, paper-based documents had to be stored on-site. After the decommissioning of FRC-Rugenbarg, all EMR were stored at the UKE due to statutory storage obligations. The data set analysed here encompasses the entire project period from the 4th of November 2015 to 21st of July 2016 and includes anonymized EMR of all patients. Before data analysis, the EMR were de-identified. Patient consent was not obtained as anonymized process data were analysed retrospectively. The local ethics committee of the Hamburg Medical Association approved the study (Ref. No. WF-053/18; 30/10/2018).

### Extraction of episodes of care

ICPC-2 was published by the WONCA (World Organization of Family Physicians) International Classification Committee (WICC) as a tool to analyse the features of primary care in the format of EOC [18]. An EOC summarizes the health care provided to a patient regarding a medical problem within a specific period of time [19, 20]. For the extraction of EOC, the study investigators (DS, MJ, and JHO) assigned ICPC-2 codes for diagnoses, complaints, and processes based on a review of all consultations of a patient. Each consultation was marked as part of one or more EOC depending on the number of medical problems that were addressed. A medical problem that was not re-addressed in later consultations was marked an EOC with a duration of 1 day. If a medical problem was addressed at more than one consultation, all related encounters were marked as part of the same EOC. If the diagnoses differed within the same EOC, we used the diagnosis of the last consultation as the principal diagnosis. Due to the heavy workload, there was no obligation for the GPs to code in ICD-10. Therefore, ICD-10 codes could be found in only 32.2% of the EMR entries. We used mapping tables obtained from the WICC website to translate ICD-10-codes into ICPC-2 codes [21]. The remaining diagnoses were extracted from free-text entries.

### Outcomes, socio-demographic data, and predictor variables

The socio-demographic data of the patients include age, gender, and country of origin. The countries of origin were grouped according to their geographical region.

The main outcome of the inferential analyses was the duration of the EOC and the provision of continuous care. There is no single definition for continuous primary care in the literature [16]. Many chronic conditions are re-evaluated by physicians every 4 weeks or every 3 months. Due to the patient fluctuation at the FRC and the rather short observation period in our study, we defined the need for continuous primary care as an EOC with a length of ≥28 days to reach adequate sensitivity for our statistical analysis. The main predictor variables for continuous care were the patients’ diagnoses. To avoid the exclusion of infrequent diagnoses for inferential statistics we generated diagnosis groups of ICPC-2-diagnoses with a prevalence < 1.0% on the episode-of-care level. Our priority was to keep single diagnoses in the data set if they fulfilled the criterion of a prevalence ≥1.0%. A second priority was a combination of a single organ system and a single diagnosis category. A third priority was the complete organ system, fourth priority was to generate combinations of organ systems. The diagnosis groups were not allowed to overlap. Dental problems were grouped into a separate diagnosis group because they are usually not treated by primary care physicians in Germany. As there were only very few congenital anomalies and neoplasms in the data set, these diagnosis categories were included in the category “other diagnoses.” Due to the wide range of episode duration of EOC with more than one visit (2 days to 251 days), a logarithmic x-scale was depicted in Figure S1.

### Data analysis

Descriptive data were presented as means and standard deviations and as percentages, respectively. We compared patients with and without continuous care by t-tests and chi^2^-tests in case of categorical variables. Socio-demographic data are shown on the patient level. Diagnoses and procedures performed by the GPs were analysed on the EOC-level. Inferential statistics were conducted by Firth’s logistic regression analyses which provide bias-reduced maximum likelihood estimates [22]. In this analysis we compared patients with and without the need for continuity of care (dependent variable). We used a multivariable model in which all diagnosis groups are represented as independent variables and each diagnosis therefore is adjusted for the influence of all other diagnoses. Additionally, the analysis was also controlled for age, gender and country of origin. Odds ratios represent the relative chance that patients with the respective diagnosis group received continuous care defined as at least 28 days of primary care treatment within our walk-in clinic. We defined an α-level of 5% (i.e., *p* ≤ 0.05) as statistically significant. Calculations were performed using Stata version 15.1.

## Results

Between the 4th of November 2015 and 21st of July 2016, there were 6219 consultations of 1516 patients at the PCWC. Due to missing EMR entries, 674 consultations (10.8%) could not be analysed. After excluding these consultations, the final data set included 5547 consultations and 4006 EOC of 1467 patients.

Sociodemographic data are shown in Table 1. Patients who had at least one EOC with a duration of ≥ 28 days were significantly older and more likely of female gender, but no significant difference was found regarding their country of origin. Adult women were on average 3 years older (adult women: 33.3 yrs. vs. adult men: 30.1 yrs). Resident reports showed that 72% of male residents were single men but just 25% were single women.

**Table 1:** Sociodemographic data and health care utilization (patient-level)

	Total	EOC^a^ < 28 days	EOC^a^ ≥ 28 days	***p***
**Age at first consultation:**				
**mean ± standard deviation;**	**23.2 ± 14.8 years;**	**22.1 ± 14.3 years**	**27.9 ± 16.2 year**	**< 0.001**
**range**	**0–73 years**	**0–73 years**	**0–71 years**	
	(*n* = 1431)	(*n* = 1145)	(*n* = 289)	
**Sex**				
**female**	**37.3%**	**35.1%**	**45.7%**	**0.001**
**male**	**62.7%**	**64.9%**	**54.3%**	
	(*n* = 1418)	(*n* = 1127)	(*n* = 291)	
country of origin^b^				
Central Asia, thereof:	46.9%	47.2%	45.6%	0.626^c^
*Afghanistan*	*46.7%*	*47.0%*	*45.6%*	
The Middle East and Northern Africa, thereof:	43.0%	43.3%	41.8%	
*Syria*	*22.3%*	*22.6%*	*21.4%*	
*Iraq*	*13.1%*	*13.1%*	*13.3%*	
*Iran*	*6.8%*	*6.9%*	*6.3%*	
Eastern, Western and Central Africa, thereof:	4.5%	4.2%	5.3%	
*Eritrea*	*2.7%*	*2.7%*	*2.8%*	
*Somalia*	*1.1%*	*0.8%*	*2.1%*	
Eastern-Central and South-Eastern Europe, thereof:	2.9%	2.7%	3.9%	
*Macedonia*	*1.0%*	*0.8%*	*1.8%*	
Post-Soviet Eurasia, thereof:	2.7%	2.5%	3.5%	
*Russia*	*2.3%*	*2.2%*	*2.8%*	
	(*n* = 1393)	(*n* = 1108)	(*n* = 285)	

^a^Episode of care (EOC); ^b^ countries < 1% are not individually shown;

^c^ groups of countries used as the unit of comparison

**Fig. 1:**
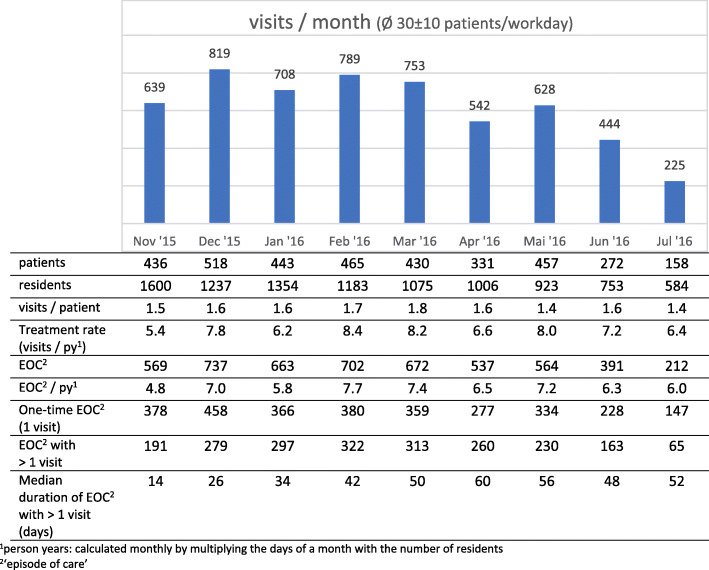
Utilization of the primary care walk-in clinic

Our data on the utilization of the PCWC are depicted on Fig. 1. In the first 7 months we observed no obvious trend in the number of encounters per month. In the last 2 months, when a decommissioning of *FRC-Rugenbarg* had started, we noted a decreased utilization due to fewer remaining residents. The monthly treatment rates and visits per patient did not decrease accordingly.

On the patient level, about 52.4% of the patients had only one physician-patient encounter at the PCWC. Another 19.8% of the patients had at least one episode with a duration of 28 days or more, which we defined as a health problem with a need for continuous primary care. The mean number of consultations per patient was 3.8 ± 3.6. EOC that were generated from these consultations averaged 2.8 ± 2.1 per patient indicating follow-up consultations within EOCs.

On the EOC level, we observed 27.8% EOC with at least one follow-up visit (e.g. EOC with a duration of > 1 day) but the majority of all EOC (72.2%) consisted of only a single consultation. A more detailed depiction of the duration of EOC by the number of consultations can be found in Figure S1 in the additional files. Not surprisingly, the mean number of consultations seems to increase with the duration of the episodes.

Table 2 depicts the distribution of EOC according to the diagnostic categories of ICPC-2 and the most common principal diagnoses of each category. The highest proportion of EOC was related to ‘infections’ (44.0%). One-fifth of the EOC could be summarized in the ICPC-2-category ‘other diagnoses’ (22.2%). Another one-fifth of all EOC was assigned to non-definitive diagnoses: ‘symptoms/complaints’ (17.1%) or ‘no disease’ (3.0%). Only 5.7% of all EOC was assigned to the ICPC-2-category “injuries.” Under routine circumstances, GPs in Germany are not consulted with dental problems. Therefore, we summarized all dental symptoms and diseases in a separate diagnosis category (6.6%). Only a few neoplasms (0.7%) were found, and congenital anomalies were also rare (0.4%).

**Table 2:** Common principal diagnoses in relation to ICPC-2 categories (episodes of care-level)

**‘Infections’** ***n*** **= 1750 (44.0% of all EOC)**	percentage of all ‘infections’
Upper respiratory tract infection (*n* = 719)	41.1%
Acute tonsillitis (n = 141)	8.1%
Gastroenteritis; presumed infection (*n* = 62)	3.5%
Acute bronchitis / bronchiolitis (*n* = 62)	3.5%
Pediculosis / other skin infestation (*n* = 60)	3.4%
Acute / chronic Sinusitis (*n* = 56)	3.2%
Viral disease other / NOS (*n* = 51)	2.9%
Cystitis / other urinary infection (*n* = 49)	2.8%
Acute otitis media / myringitis (*n* = 40)	2.3%
Scabies / other ascariasis (*n* = 37)	2.1%
**‘Other diagnoses’** ***n*** **= 887 (22.2% of all EOC)**	percentage of all ‘other diagnoses’
Stomach function disorder (*n* = 69)	7.8%
Back syndrome w/o radiating pain (*n* = 48)	5.4%
Dermatitis / atopic eczema (*n* = 47)	5.3%
Acne (*n* = 27)	3.0%
Depressive disorder (*n* = 26)	2.9%
Health maintenance / prevention (*n* = 25)	2.8%
Excessive ear wax (*n* = 23)	2.6%
Asthma (*n* = 22)	2.5%
Skin disease, other (*n* = 21)	2.3%
Tension-type headache (n = 21)	2.3%
**‘Symptoms/Complaints’** ***n*** **= 683 (17.1% of all EOC)**	Percentage of all ‘Symptoms/Complaints’
Constipation (*n* = 46)	6.7%
Headache (n = 40)	5.6%
Throat symptom / complaint (n = 25)	3.6%
Knee symptom / complaint (n = 25)	3.6%
Diarrhoea (*n* = 18)	2.6%
Heartburn (n = 17)	2.5%
Cough (n = 17)	2.5%
Rash localized (n = 17)	2.5%
Fever (*n* = 16)	2.3%
**‘Injuries’** ***n*** **= 228 (5.7% of all EOC)**	Percentage of all ‘injuries’
Abrasion / scratch / blister (n = 29)	12.7%
Sprain/strain of joint NOS (n27)	11.8%
Sprain/strain of ankle (n = 22)	9.6%
Injury musculoskeletal NOS (n = 18)	7.9%
Burn/scald (*n* = 15)	6.6%
Laceration / cut (n = 13)	5.7%
Other Skin injury (n = 13)	5.7%
Trauma / injury NOS (*n* = 9)	3.9%
Insect bite / sting (n = 8)	3.5%
Fracture: other location (n = 6)	2.6%

‘No disease’ (*n* = 121) (3.0% of all EOC)

‘Dental symptoms/disease’ *n* = 265 (6.6% of all EOC)

In EOC with a duration of ≥ 28 days, there were less infections (32.1% vs. 47.5%; *p* < 0.001), but more EOC from the ICPC-2-categories “other diagnoses” (61.4% vs. 29.5%; *p* < 0.001), symptomatic diagnoses (29.9% vs. 20.2%; p < 0.001), neoplasms (2.2% vs. 0.6%; *p* = 0.001) and congenital anomalies (1.6% vs. 0.3%; p < 0.001). The prevalence of “upper respiratory infection, acute” was significantly lower (11.7% vs. 21.5%; *p* < 0.001) in EOC ≥ 28 days, but there were more diagnoses of “no disease” (6.0% vs. 3.2%; *p* = 0.005) and a much higher prevalence of “dermatitis/atopic eczema” in EOC with EOC ≥ 28 days (3.8% vs. 1.0%; p < 0.001).

**Fig. 2:**
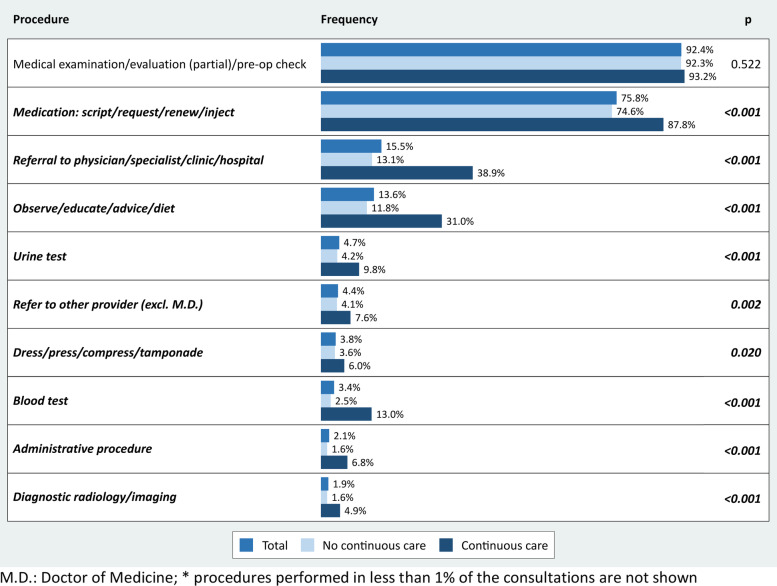
ICPC-2 procedures* (episodes of care-level)

The procedures (according to ICPC-2) that were performed by the GPs are shown in Fig. 2. More than 9 out of 10 episodes included a (partial) medical examination, about 3 out of 4 episodes resulted in a medication and in 3 out of 20 episodes, the GPs initiated a referral to an outpatient specialist or a hospital. The largest differences between episodes with and without continuous care were found in the utilisation of blood tests (5.2 times higher utilisation in continuous care episodes), administrative procedures (4.3), diagnostic radiology/imaging (3.1) and referrals to secondary care (3.0).

**Fig. 3:**
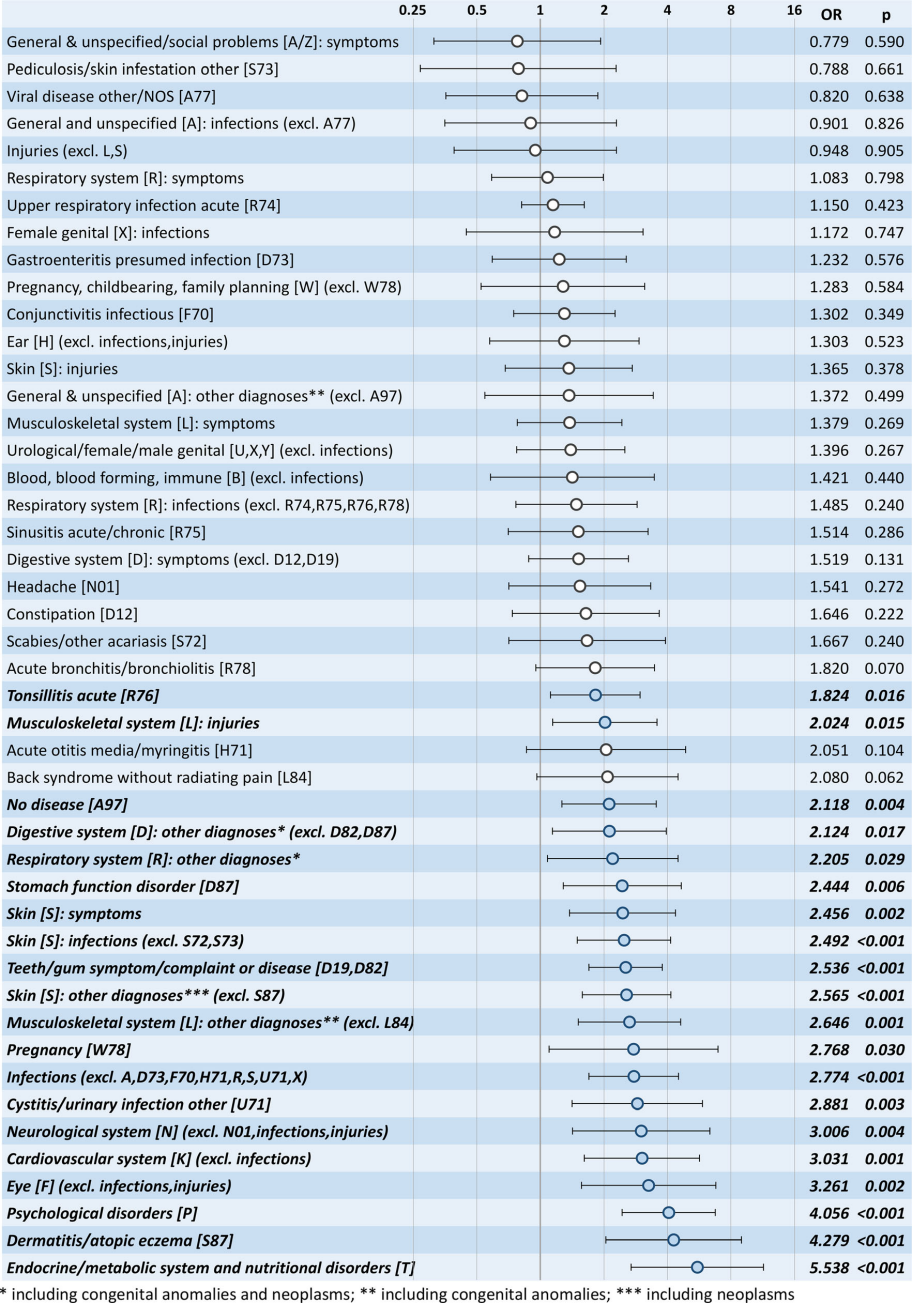
Association of diagnosis groups with continuous primary care: results from logistic Firth regression analyses controlling for age, gender and country of origin

The association between the diagnosis groups and the need for continuous primary care of ≥ 28 days is shown in Fig. 3. A total of 20 of the 46 diagnosis groups showed an association with EOC ≥ 28 days. The highest effect was found in the diagnosis groups “psychological disorders”, “dermatitis/atopic eczema,” and “endocrine/metabolic system and nutritional disorders.”

## Discussion

### Principal findings

We observed a high utilization rate at the PCWC reflected by monthly treatment rates between 5.4 and 8.0 visits per personyear (py) and an average of 30 patient visits per working day. Infectious diseases were treated in more than 40% of all EOC and non-communicable diseases in more than one fifth of EOC. Remarkably, in another one fifth of EOC, GPs provided only symptomatic diagnoses or noted ‘no disease’. Taking into account that only 3% of all EOC were attributed to psychological diagnoses, we assume that the prevalence of mental health diseases was underestimated in the investigated care model.

Most camp residents needed only single GP visits to deal with their health problems reflecting the low prevalence of severe or other chronic diseases in a young population. However, we observed that nearly one fifth of camp residents had EOC that lasted for more than 4 weeks which we interpreted as demand for continuous primary care in this subgroup. In EOC with continuous care we observed significantly more imaging and laboratory testing as well as 2.5 times more referrals to secondary care. Patients of this subgroup were significantly older and more likely of female gender. We propose that even in a young population of asylum seekers a subgroup of patients showed a demand for continuity of care, care coordination and more resources (e.g. imaging, laboratory tests, referrals). Typical chronic diseases were the strongest predictors for the demand of continuous care.

### Strength and limitations of the study

One strength of the investigated care model was that low barriers existed for the GPs to utilize secondary care services. When diagnostic imaging, laboratory testing or referral to secondary care were deemed to be medically necessary within an EOC, it could be initiated by the GPs without any additional costs. Hence, we assume that no formal or financial barriers existed that could have encouraged GPs to withhold from utilization of these services. Therefore, we assume that the proportion of initiated procedures seem to reflect the medical needs rather than procedural barriers.

Regarding the applicability of our data we note that virtually all published studies on health care at refugee camps provided consultation-based data that use given diagnoses of a consultation as a proxy of disease prevalence [8, 23]. The expenditure of time and workforce may be underestimated through such a consultation-based analysis. The strength of our analysis is that multiple medical complaints of a single consultation were resolved in different EOC, and follow-up consultations were assigned to the same EOC. We believe our EOC-based approach improves the quality and applicability of our data in terms of care planning.

A limitation of our study is that the extraction of EOC was retrospective and mainly based on free-text EMR entries. However, ambiguous cases were regularly discussed and solved by the consent of the three investigators.

Another limitation is that the analysed population was changing due to newly arriving asylum seekers. However, we documented 90 EOC with a duration of more than 100 days. Furthermore, the distribution of EOC with > 1 visit and the median duration of these EOC was after the first 2 months between 34 and 60 days. Therefore, we could show that a relevant number of residents stayed for several months.

Our statistical analyses were adjusted for age, gender and country of origin. Due to the limited data set, it was not possible to consider other factors like the duration of the journey, trauma experience, social support, educational background and financial situation. All of these factors could have an influence on the disease spectrum and the need for continuous care.

### Comparison with existing literature

#### Utilization of the primary care service

The mean age, as well as the gender ratio and percentage of minors in our population, matched the demographics of 2753 patients at a refugee clinic in eastern Germany in 2015 as well as of 2291 refugees at a long-stay refugee camp on Lesbos in 2016 [8, 10]. Based on monthly resident reports we could estimate a mean percentage of 71% male residents in the camp. Considering the percentage of 62.7% males in our study, we believe that our data indicates a poorer health seeking behaviour of male residents which is in line with the existing literature [24].

In comparison with the outpatient clinic of the UNHCR Za’atri long-stay refugee camp in Jordan (over 79.000 residents), we had the same rate of consultations per physician (30 per working day) at five working days per week. Our mean monthly treatment rate (TR) of 7.1 was higher than the TR of 6.5 at the Za’atri camp [25]. According to the sphere standard, a TR of 2–3 – which is much lower than in our study – can be expected in a disaster-affected population [26]. Use of primary care services is reported to be generally higher among migrants compared to native patients [6]. This observation was frequently explained with inadequate access to secondary care services. However, in our setting there were merely financial nor procedural barriers for referrals to secondary care and the TR was relatively high. Although the GPs at the PCWC were provided with access to an online interpreter, there was no obligation to document its use in the EMR. Therefore, we are not able to determine how frequent interpreters were used. Remarkably, it can be found in the literature that GPs generally emphasize the importance of language barriers but rarely make use of available formal interpreters [27, 28]. In the PCWC GPs saw, on average, 30 patients per day. Supposedly, all of them with a language barrier. It was not possible to use online interpreters in every consultation. Different studies showed that cross-cultural consultations that end without mutual understanding result in patients keep on coming back to resolve their health and social care needs [27]. Therefore, we conclude that a low-threshold primary care service with access to formal interpreters can still not overcome all barriers of cross-cultural consultation between GPs and migrants.

The highest amount of provided medical care was related to infections, which is in line with most cross-sectional observations [14, 23]. Non-communicable diseases (ICPC-2 category ‘other diagnoses’) comprised one-fifth of provided medical care. We noted that another one-fifth of all EOC had symptomatic diagnoses or ‘no disease’ as a principal diagnosis. This proportion is consistent with the estimated high number of medically unexplained symptoms (MUS) in primary care [29, 30]. MUS are reported to be more common among women, younger age groups, and patients with a lower socioeconomic background [31] which reflects our study population. Remarkably, MUS are associated with the presence of psychiatric disorders. We therefore suppose that in our study the amount of symptomatic diagnoses can be regarded as an indicator for missed diagnoses of mental health disorders. The PCWC was utilized by patients to articulate not only medical problems but also administrative and social problems. We note that the ICPC-2 code ‘administrative procedure’ was attributed to 2.1% of EOC. This could have caused the GPs to assign symptomatic diagnoses or note ‘no disease’ which was a predictor for longer care episodes in our analysis. It could be shown that GPs in a routine care setting were reluctant to diagnose a suspected underlying psychosomatic cause for the expressed complaints [32]. Therefore, the relatively low amount of mental health problems in our sample is likely an underestimation. Studies that used screening instruments for mental health problems estimated a much higher prevalence of depression, PTSD, and somatisation in asylum seekers [5, 33]. Although the investigated PCWC was deemed “low-threshold” in terms of availability of GPs and interpreters, we propose that personal barriers of refugee seekers could also play a role. It was shown that besides language barriers and cultural differences, stigma, a lack of awareness of services and different expectations between patient and provider can lead to underutilisation of mental health services by asylum seekers [34, 35].

#### Demand for continuous care and care coordination

Patients with a demand for continuous care were older and more likely of female gender. An explanation could be that female residents that were much more likely caring for children showed a stronger health seeking behavior.

We observed the strongest association of diagnosis groups to continuous primary care with typical chronic diseases. But there was also an association of acute conditions like tonsillitis and urinary tract infections with longer care episodes. Taking the young average age of our sample into account, this seems even more surprising. An explanation could be that the housing conditions in terms of hygiene standards and crowding (more than 1000 individuals under one roof) led to an increased risk for prolonged common infections and relapses.

In continuous care episodes medical procedures were utilised more often than in episodes with a shorter duration. The main reasons for these differences are probably the larger number of contacts with the primary care physicians during continuous care episodes. However, some services had a disproportionally high utilisation in episodes of continuous care, including blood tests, administrative procedures (sick certificates, attestations, certificates, etc.), imaging and referrals. This marks the continuous care episodes as key episodes to estimate the demand for coordination of care by GPs. On the other hand, these episodes could be further analysed whether a potential for overtreatment and unnecessary healthcare expenditures exist. In particular, referral rates are considered an important determinant of secondary care utilization. Reported referral rates were between 10 and 13% of physician-patient encounters in the US and Norway [36, 37]. In our sample, we noted a higher referral rate of 15.5%. However, compared to these studies, we used a different approach by calculation of the referral rate with the EOC (which could consist of more than consultation) and not the consultation as a denominator. We regard this as a better estimation of secondary care utilization because we can estimate referral rates based on the health problem (e.g., EOC), rather than on the total number of consultations. In primary care-based health systems such as those in the Netherlands and Norway, there is a requirement of a GP-visit before accessing specialty care. It could be shown that this ‘gatekeeping’ is associated with lower health care expenditures on ambulatory care [38, 39]. On the other hand, it was proposed that restricted access to medical care for refugees could even increase health care expenditures in Germany [40]. Although there is no gatekeeping for regular patients in Germany, we could show that under circumstances of a factual gatekeeping at the camp’s PCWC, GPs were able to treat the majority of health problems without referrals.

### Implications for clinical practice

The investigated PCWC with online access to professional interpreters on-site represented a probably convenient low-threshold care service for the residents as the high treatment rates indicate. This led to a challenging workload for the GPs.

Our data implicates that a number of mental health issues was probably masked behind symptomatic diagnoses. Therefore, a systematic screening for mental health disorders remains indispensable and could not be compensated in the investigated care model.

The observed need for care continuity, care coordination and management of symptomatic complaints without a definitive diagnosis should be recognized by stakeholders of future care planning for asylum seekers. Paradigms of emergency care that are mainly consultation based and concentrate on treatment of a single acute complaint maybe not sufficient.

To ensure effective care continuity and care coordination the use of EMR that allows the recognition of care episodes by linkage of single consultations is strongly recommended.

## Conclusions

The utilization of ICPC-2 in combination with the concept of EOC turned out to be a valuable tool for the investigation of primary care provision at a refugee camp. A wide spectrum of acute and chronic health conditions could be treated by GPs without referrals or the use of other medical resources. However, a systematic screening for mental health disorders is recommended in this setting.

Principles of primary care like care continuity, care coordination and management of symptomatic complaints should be emphasized in future care planning for refugee camps.

## Supplementary information

**Additional file 1: Figure S1.** Duration of episodes of care by number of consultations* (episodes of care-level).

## Data Availability

The datasets used and/or analysed during the current study are available from the corresponding author on reasonable request.

## Abbreviations

EMR: Electronic medical records
EOC: Episodes of care
EU: European Union
FRC: First Reception Centre
GP: General practitioner
WICC: International Classification Committee
ICPC-2: International classification of primary care-2nd edition
MUS: Medically unexplained symptoms
py: Personyear
PTSD: Post-traumatic Stress Disorder
PCWC: Primary Care walk-in clinic
TR: Treatment rate
UKE: University Medical Center Hamburg-Eppendorf
WONCA: World Organization of Family Physicians

## References

[1] “Das Bundesamt in Zahlen 2015” – Publication – Federal Office for Migration and Refugees 2015. http://www.bamf.de/SharedDocs/Anlagen/DE/Publikationen/Broschueren/bundesamt-in-zahlen-2015.html. Accessed 29 Jan 2020.

[2] “Das Bundesamt in Zahlen 2016” – Publication – Federal Office for Migration and Refugees 2016. https://www.bamf.de/SharedDocs/Anlagen/DE/Publikationen/Broschueren/bundesamt-in-zahlen-2016.html. Accessed 29 Jan 2020.

[3] Data regarding migration – Daten zur Zuwanderung, Zentraler Koordinierungsstab Flüchtlinge (ZKF), Hamburg. hamburg.de. https://www.hamburg.de/fluechtlinge-daten-fakten/. Accessed 29 Jan 2020.

[4] Bozorgmehr K, Mohsenpour A, Saure D, Stock C, Loerbroks A, Joos S (2016). Systematic review and evidence mapping of empirical studies on health status and medical care among refugees and asylum seekers in Germany (1990-2014). Bundesgesundheitsblatt Gesundheitsforschung Gesundheitsschutz.

[5] Nesterko Y, Jäckle D, Friedrich M, Holzapfel L, Glaesmer H (2019). Prevalence of post-traumatic stress disorder, depression and somatisation in recently arrived refugees in Germany: an epidemiological study. Epidemiol Psychiatr Sci.

[6] Bauhoff S, Göpffarth D (2018). Asylum-seekers in Germany differ from regularly insured in their morbidity, utilizations and costs of care. PLoS One.

[7] Puchner K, Karamagioli E, Pikouli A, Tsiamis C, Kalogeropoulos A, Kakalou E (2018). Time to rethink refugee and migrant health in Europe: moving from emergency response to integrated and individualized health care provision for migrants and refugees. IJERPH..

[8] Goodman LF, Jensen GW, Galante JM, Farmer DL, Taché S (2018). A cross-sectional investigation of the health needs of asylum seekers in a refugee clinic in Germany. BMC Fam Pract.

[9] Ackermann N, Marosevic D, Hörmansdorfer S, Eberle U, Rieder G, Treis B (2018). Screening for infectious diseases among newly arrived asylum seekers, Bavaria, Germany, 2015. Euro Surveill.

[10] Hermans MPJ, Kooistra J, Cannegieter SC, Rosendaal FR, Mook-Kanamori DO, Nemeth B (2017). Healthcare and disease burden among refugees in long-stay refugee camps at Lesbos, Greece. Eur J Epidemiol.

[11] Pavli A, Maltezou H. Health problems of newly arrived migrants and refugees in Europe. J Travel Med. 2017;24. 10.1093/jtm/tax016. 10.1093/jtm/tax016 28426115

[12] van Berlaer G, Bohle Carbonell F, Manantsoa S, de Béthune X, Buyl R, Debacker M (2016). A refugee camp in the Centre of Europe: clinical characteristics of asylum seekers arriving in Brussels. BMJ Open.

[13] Doocy S, Lyles E, Roberton T, Akhu-Zaheya L, Oweis A, Burnham G (2015). Prevalence and care-seeking for chronic diseases among Syrian refugees in Jordan. BMC Public Health.

[14] Alberer M, Wendeborn M, Löscher T, Seilmaier M (2016). Spectrum of diseases occurring in refugees and asylum seekers: data from three different medical institutions in the Munich area from 2014 and 2015. Dtsch Med Wochenschr.

[15] Nutting PA, Goodwin MA, Flocke SA, Zyzanski SJ, Stange KC (2003). Continuity of primary care: to whom does it matter and when?. Ann Fam Med.

[16] Kringos DS, Boerma WG, Hutchinson A, van der Zee J, Groenewegen PP (2010). The breadth of primary care: a systematic literature review of its core dimensions. BMC Health Serv Res.

[17] Refugee First Response Center. https://refugeefirstresponsecenter.com/. Accessed 29 Jan 2020.

[18] Lamberts H, Wood M (2002). The birth of the international classification of primary care (ICPC) serendipity at the border of lac Léman. Fam Pract.

[19] Hussey PS, Friedberg MW, Anhang Price R, Lovejoy SL, Damberg CL (2017). Episode-based approaches to measuring health care quality. Med Care Res Rev.

[20] Soler J-K, Okkes I, Wood M, Lamberts H (2008). The coming of age of ICPC: celebrating the 21st birthday of the international classification of primary care. Fam Pract.

[21] The International Classification of Primary Care (ICPC) 2nd edition. ehelse.no. https://ehelse.no/icpc-2e-english-version. Accessed 29 Jan 2020.

[22] Firth D (1993). Bias reduction of maximum likelihood estimates. Biometrika..

[23] Borgschulte HS, Wiesmüller GA, Bunte A, Neuhann F (2018). Health care provision for refugees in Germany – one-year evaluation of an outpatient clinic in an urban emergency accommodation. BMC Health Serv Res.

[24] Bhatia R, Wallace P (2007). Experiences of refugees and asylum seekers in general practice: a qualitative study. BMC Fam Pract.

[25] The UN Refugee Agency. Detailed health indicator report, Za’atri refugee camp, Jordan. 2016. https://data2.unhcr.org/en/documents/details/52185. Accessed 29 Jan 2020.

[26] The Sphere Project (2011). Humanitarian charter and minimum standards in humanitarian response: the sphere handbook.

[27] van den Muijsenbergh M, van Weel-Baumgarten E, Burns N, O’Donnell C, Mair F, Spiegel W (2014). Communication in cross-cultural consultations in primary care in Europe: the case for improvement. The rationale for the RESTORE FP 7 project. Prim Health Care Res Dev.

[28] Papic O, Malak Z, Rosenberg E (2012). Survey of family physicians’ perspectives on management of immigrant patients: attitudes, barriers, strategies, and training needs. Patient Educ Couns.

[29] Soler JK, Okkes I (2012). Reasons for encounter and symptom diagnoses: a superior description of patients’ problems in contrast to medically unexplained symptoms (MUS). Fam Pract.

[30] Steinbrecher N, Koerber S, Frieser D, Hiller W (2011). The prevalence of medically unexplained symptoms in primary care. Psychosomatics..

[31] Nimnuan C, Hotopf M, Wessely S (2001). Medically unexplained symptoms: an epidemiological study in seven specialities. J Psychosom Res.

[32] Pohontsch NJ, Zimmermann T, Jonas C, Lehmann M, Löwe B, Scherer M (2018). Coding of medically unexplained symptoms and somatoform disorders by general practitioners – an exploratory focus group study. BMC Fam Pract.

[33] Belz M, Belz M, Özkan I, Graef-Calliess IT (2017). Posttraumatic stress disorder and comorbid depression among refugees: assessment of a sample from a German refugee reception center. Transcult Psychiatry.

[34] Satinsky E, Fuhr DC, Woodward A, Sondorp E, Roberts B (2019). Mental health care utilisation and access among refugees and asylum seekers in Europe: a systematic review. Health Policy.

[35] Shannon PJ, Wieling E, Simmelink-McCleary J, Becher E (2015). Beyond stigma: barriers to discussing mental health in refugee populations. J Loss Trauma.

[36] Ringberg U, Fleten N, Deraas TS, Hasvold T, Førde O (2013). High referral rates to secondary care by general practitioners in Norway are associated with GPs’ gender and specialist qualifications in family medicine, a study of 4350 consultations. BMC Health Serv Res.

[37] Barnett ML, Song Z, Landon BE (2012). Trends in physician referrals in the United States, 1999-2009. Arch Intern Med.

[38] Delnoij D, Van Merode G, Paulus A, Groenewegen P (2000). Does general practitioner gatekeeping curb health care expenditure?. J Health Serv Res Policy.

[39] Garrido MV, Zentner A, Busse R (2011). The effects of gatekeeping: a systematic review of the literature. Scand J Prim Health Care.

[40] Bozorgmehr K, Razum O (2015). Effect of restricting access to health care on health expenditures among asylum-seekers and refugees: a quasi-experimental study in Germany, 1994–2013. PLoS One.

