# Supplementary material for: Episodes of care in a primary care walk-in clinic at a refugee camp in Germany – a retrospective data analysis
Source: BMC Fam Pract. 2020 Sep 21;21:193. doi: 10.1186/s12875-020-01253-3 (PMC7507675; doi:10.1186/s12875-020-01253-3)
Supplement: Supplementary file 1 — Additional file 1: Figure S1. Duration of episodes of care by number of consultations* (episodes of care-level). [file 12875_2020_1253_MOESM1_ESM.docx]

**Episodes of care in a primary care walk-in clinic at a refugee camp in Germany– a retrospective data analysis**

**Supplement**

Jan Hendrik Oltrogge, Ingmar Schäfer, Dana Schlichting, Martin Jahnke, Anja Rakebrandt, Susanne Pruskil, Hans-Otto Wagner, Dagmar Lühmann, Martin Scherer

**Figure S1: Duration of episodes of care by number of consultations* (episodes of care-level)**


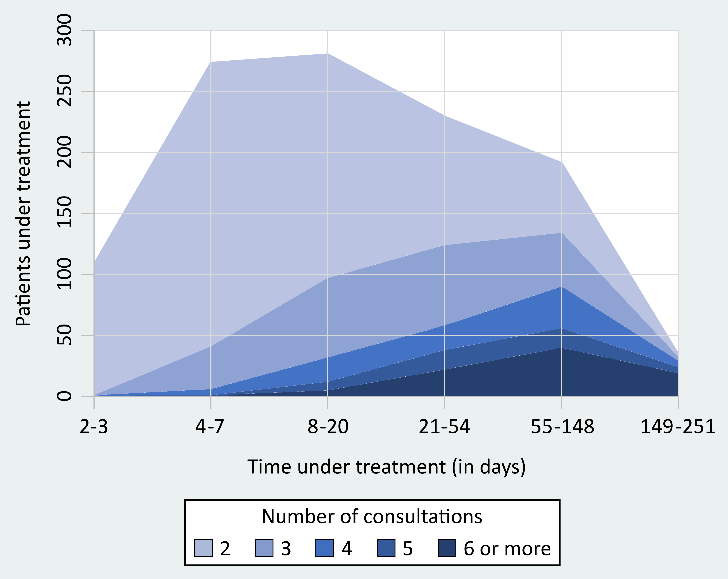


* episodes of care with a duration of one day (72.5% of all episodes) have been excluded
